# Comparison of anti-PD-1/PD-L1-based regimens in relapsed/refractory diffuse large B-cell lymphoma: a meta-analysis

**DOI:** 10.7717/peerj.20314

**Published:** 2025-11-19

**Authors:** Wenxin Jiang, Tingyu Wen, Peng Liu

**Affiliations:** National Cancer Center/National Clinical Research Center for Cancer/Cancer Hospital, Chinese Academy of Medical Sciences & Peking Union Medical College, Chaoyang District, Beijing, China

**Keywords:** Diffuse large B-cell lymphoma, Programmed death 1, Programmed death ligand 1, Immune checkpoint inhibitors, Immunotherapy

## Abstract

**Aim:**

To evaluate the therapeutic value and find out potential combination agents of programmed death 1/programmed death-ligand 1 (PD-1/PD-L1) monoclonal antibody (mAb) in relapsed/refractory (r/r) diffuse large B-cell lymphoma (DLBCL).

**Methods:**

We conducted a meta-analysis to assess the efficacy and safety of PD-1/PD-L1 mAb in r/r DLBCL, potential qualified studies were searched in PubMed, Embase, Web of Science, and ClinicalTrials.gov. This meta-analysis had been registered on the PROSPERO platform (CRD42023340031).

**Results:**

After systematic screening, a total of 32 records involving 29 studies were included, pooled survival curves indicated better progression-free survival (PFS) (*p* < 0.0001; HR = 0.51, 95% CI [0.42–0.62]) and overall survival (OS) (*p* = 0.013; HR = 0.71, 95% CI [0.57–0.88]) for combination therapy compared with monotherapy. Combination therapy group also achieved a better pooled complete response rate (CRR) (14.6% *vs.* 3.0%; *p* < 0.001) and overall response rate (ORR) (30.5% *vs.* 10.3%; *p* < 0.001). Analysis of the incidence of adverse events (AEs) did not demonstrate additional toxicities of combination therapy. The limitation was the predominance of single-arm trials, precluding the direct comparison of combination versus partner agents alone.

**Conclusions:**

These findings support further exploration of PD-1/PD-L1-mAb-based combination therapy to identify long-term survival benefits, while application of monotherapy in unselected DLBCL patients is not recommended.

## Introduction

As the most common subtype of non-Hodgkin lymphoma (NHL), diffuse large B-cell lymphoma (DLBCL) accounts for approximately 20% of all lymphomas (Teras et al., 2016). Although first-line R-CHOP treatment (rituximab, cyclophosphamide, anthracyclines, vinblastine, and prednisone) can achieve a cure rate of 50–60%, 20% of patients have primary refractory disease, and 30% relapse after complete response (CR) (Coiffier & Sarkozy, 2016). The phase III POLARIX study reported a superior progression-free survival (PFS) of the polatuzumab vedotin–rituximab–cyclophosphamide–doxorubicin–prednisone (Pola-R-CHP) regimen compared with R-CHOP (Tilly et al., 2022), which has changed the first-line treatment paradigm for DLBCL, although an overall survival (OS) benefit has not yet been demonstrated. Salvage chemotherapy followed by autologous stem cell transplantation (ASCT) could provide an opportunity for such patients, however, only half are eligible for transplantation due to age and comorbidities, and about 50% of the eligible patients can execute ASCT after response of salvage chemotherapy. As a result, the final cure rate remains only 25–35% (Crump et al., 2014; Gisselbrecht et al., 2010). Although chimeric antigen receptor T-cell (CAR-T) therapy demonstrate a significant clinical benefit (Abramson et al., 2020; Schuster et al., 2019), its application is restricted by economic conditions and accessibility (Lin et al., 2019), so are antibody-drug conjugate (ADC) and bispecific antibody (BsAb), especially in developing countries. Other targeted drugs, such as Bruton’s tyrosine kinase inhibitor (BTKi) and lenalidomide, exhibit modest activity (Wilson et al., 2015; Czuczman et al., 2017). Therefore, there is still a huge treatment demand for relapsed/refractory (r/r) DLBCL patients, particularly in those ineligible for transplantation.

Programmed death 1 (PD-1), a member of the CD28 superfamily, is an immune co-inhibitory receptor whose identified ligands include programmed death-ligand 1 (PD-L1) and programmed death-ligand 2 (PD-L2). Its immunoreceptor tyrosine based inhibition motif (ITIM) and immunoreceptor tyrosine based switch motif (ITSM) in intracellular domain can be phosphorylated by SRC proto-oncogene (SRC) family tyrosine kinase after ligand binding, and the dephosphorylation of phosphatidylinositol-3-kinase (PI3K) by downstream molecules exerts an immune suppressive effect (Isoyama et al., 2021). PD-1/PD-L1 monoclonal antibodies (mAbs) have been widely used in various malignancies (Zhang et al., 2025), and have changed their treatment patterns. DLBCL leads the highest level of PD-L1 expression in B-cell NHLs (Yang & Hu, 2019), but PD-1/PD-L1 mAbs are only utilized as a later-line therapeutic option for DLBCL, with insufficient evidence to guide their clinical application.

Based on preclinical evidence, a large number of studies are currently exploring the efficacy of PD-1/PD-L1 mAb in r/r DLBCL patients as exploratory treatments. Due to the unsatisfactory efficacy of monotherapy, multiple combination strategies are under investigation. However, the efficacy and safety of each regimen, especially long-term survival, remain unclear. This meta-analysis aimed to analyze various regimens and guide the potential clinical application of PD-1/PD-L1 mAb in r/r DLBCL.

## Methods

### Search strategy

Potential qualified studies were searched in PubMed, Embase, Web of Science, and ClinicalTrials.gov. The deadline of retrieval time was April 15, 2025. To reduce potential omissions due to the variable terminology used in DLBCL-related literature (*e.g.*, “B-cell lymphoma” or similar descriptors), we employed a broader search strategy and subsequently applied stringent criteria to exclude ineligible studies during the screening process. Taking PubMed as an example, the searching strategy was: (Programmed death ligand 1[tiab] OR PD-L1[tiab] OR Programmed death 1[tiab] OR PD-1[tiab] OR Anti-Programmed death ligand 1[tiab] OR Anti-PD-L1[tiab] OR Anti-Programmed death 1[tiab] OR Anti-PD-1[tiab] OR Atezolizumab[tiab] OR Durvalumab[tiab] OR Nivolumab[tiab] OR Pembrolizumab[tiab] OR Sintilimab[tiab] OR Tislelizumab[tiab] OR Avelumab[tiab] OR Camrelizumab[tiab] OR Toripalimab[tiab] OR Cemiplimab[tiab] OR Penpulimab[tiab] OR Sugemalimab[tiab] OR Zimberelimab[tiab]) AND lymphoma[tiab]. There was no restriction on language, region, race and age. In addition, literature reviews and references of original researches were also screened to avoid omissions. This meta-analysis had been registered on PROSPERO platform (registration ID: CRD42023340031).

### Inclusion and exclusion criteria

Inclusion criteria: (1) prospective clinical studies; (2) pathology type: DLBCL, not otherwise specified (NOS); studies focusing on B-cell lymphoma or NHL were also eligible if they reported outcomes of the DLBCL cohort; (3) primary refractory or relapsed disease after first-line therapy; (3) patients receiving PD-1/PD-L1 mAb treatment, including monotherapy and combination therapy with other agents; (4) studies reporting efficacy and safety endpoints.

Exclusion criteria: (1) prospective studies with a sample size of less than 10 patients; (2) first-line treatment researches; (3) patients with central nervous system invasion and primary mediastinal large B-cell lymphoma (PMBL); (4) researches related to ASCT; (5) pathology type: transformed follicular lymphoma, Richter syndrome, high-grade B-cell lymphoma (HGBL); (6) researches involving pidilizumab; (7) article type: retrospective study, review, comment, case report, cell or animal research.

### Quality assessment

Randomized controlled studies (RCT) were evaluated by Cochrane Collaboration’s tool for assessing risk of bias in randomized trials (Higgins et al., 2011), while non-randomized studies (including single-arm studies) were evaluated by methodological index for non-randomized studies (MINORS) (Slim et al., 2003). Two reviewers Wenxin Jiang and Tingyu Wen independently evaluated the quality of researches.

### Data extraction

Two researchers, Wenxin Jiang and Tingyu Wen, independently conducted study inclusion and data collection, the third author, Peng Liu, would discuss if there was disagreement between the two researchers. The following characteristic information of included studies was recorded: author, year of publication, study type, clinical study phase, sample size, treatment, disease status, follow-up time, median age and gender ratio. The data of the latest published literature would be adopted if there were multiple published literature for the same study. If original survival data could not be accessed, data would be extracted from Kaplan–Meier (KM) curves using software Engage Digitizer version 10.8 (Guyot et al., 2012).

### Statistical analysis

Primary endpoints included PFS and OS, secondary endpoints included overall response rate (ORR), CR rate (CRR) and incidence of adverse events (AEs) (AEs of all enrolled patients in the study would be reported if no AEs data of the DLBCL cohort was provided). Statistical analysis was conducted using STATA/SE version 18.0 on the pooled ORR, CRR, and incidences of AEs (Boston & Sumner, 2003). The pooled KM curves were estimated and analyzed using MetaSurv package of R software version 4.3.1 (R Core Team, 2023) by the product-limit estimator method (Combescure, Foucher & Jackson, 2014). All pooled effect sizes were reported with 95% confidence intervals (CI). The Cochrane Q chi square test and I^2^ statistic were applied to test the heterogeneity. Fixed effects model was applied for pooled results with low heterogeneity (I^2^ ≤ 50%), otherwise random effects model was used. Sensitivity analysis was conducted for pooled results with high heterogeneity by excluding each study one by one, and potential publication bias of included studies was examined using Begg’s test, Egger’s test and funnel plot.

## Results

### Included studies characteristics

**Figure 1:**
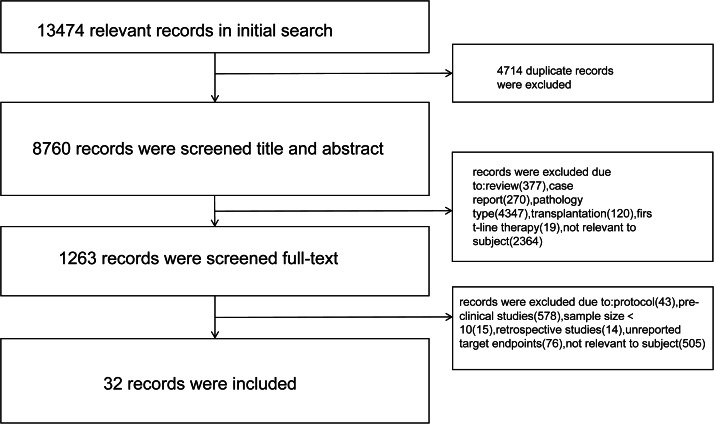
Screening process diagram.

After screening, a total of 32 records (published literature *n* = 30; records from clinicalTrials.gov *n* = 2) involving 29 studies were included. One eligible RCT, one non-randomized controlled trial, and 27 single-arm trials (including studies reporting multiple independent cohorts) were included. Screening process diagram was shown in Fig. 1, and characteristics of included studies were shown in Table 1. A total of 948 patients were included in this analysis, with 195 (20.6%) patients receiving PD-1/PD-L1 mAb monotherapy and 753 (79.4%) patients receiving combination regimens.

### Quality assessment

Quality evaluations of single-arm and non-randomized controlled studies were conducted by MINORS: literature of highest quality, CHECKMATE-139/NCT02038933 scored 16 points, other literature scored between 13–15 points (Table S1). The quality evaluation of the single RCT (ARGO/NCT03422523) was conducted by Cochrane risk bias assessment tool (Fig. S1).

### Efficacy

#### PFS

**Figure 2:**
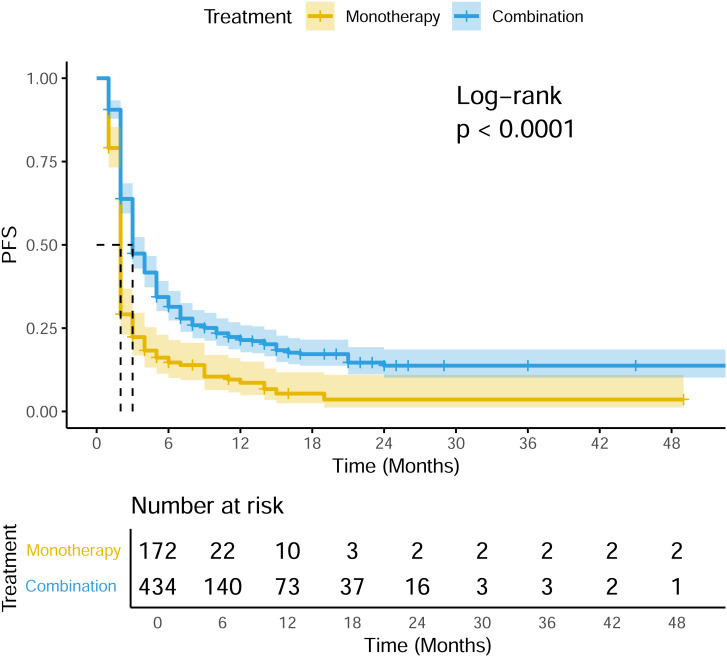
Pooled PFS curves of DLBCL patients treated with PD-1/PD-L1 mAb monotherapy and combination therapy.

**Figure 3:**
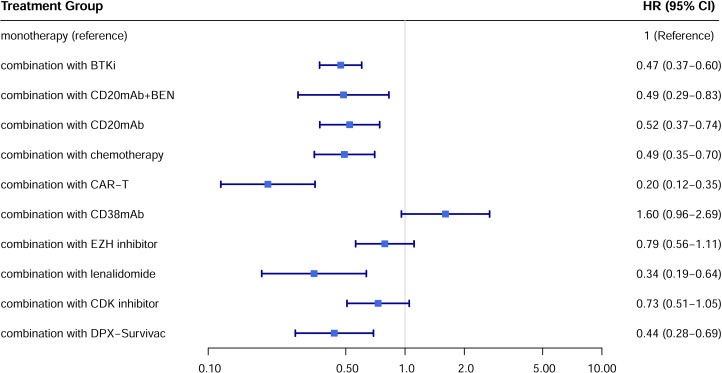
Hazard ratio for PFS of each treatment subgroup compared with monotherapy.

A total of 22 studies exhibited available PFS curves. The pooled analysis demonstrated a statistical improvement in PFS (*p* < 0.0001) of combination therapy compared with monotherapy (Fig. 2), with a hazard ratio (HR) of 0.51 (95% CI [0.42–0.62]). The 12-month PFS rates were 21.4% for combination therapy and 8.6% for monotherapy, while the 24-month PFS rates were 13.7% and 3.6%, respectively. Subgroup analysis was conducted due to the high heterogeneity, separate HR of each combination subgroup compared with monotherapy were shown in Fig. 3, the combination with CAR-T group demonstrated the most significant PFS benefit (HR 0.20; 95% CI [0.12–0.35]).

**Table 1:** Characteristics of included studies.

Year	Author	Study	Type	Phase	Follow-up	Treatment	Patients	Sample size	Median age	Male (%)
2019	Stephen M. Ansell	CHECKMATE-139/NCT02038933 Ansell et al. (2019)	single-arm	II	9 m (range: 0.1–25)	nivolumab	r/r DLBCL (ASCT failed)	87	62	64
					6 m (range: 0.2–24)		r/r DLBCL (ASCT ineligible)	34	68	62
2016	Lesokhin, A.M.	CHECKMATE-039/NCT01592370 Lesokhin et al. (2016)	single-arm	Ib	22.7 w	nivolumab	r/r DLBCL	11	n/a	n/a
2022	Elise A Chong	NCT02650999 Chong et al. (2022)	single-arm	I/IIa	n/a	pembrolizumab	r/r DLBCL (after CD19 CAR-T)	11	n/a	n/a
2022	John Kuruvilla	KEYNOTE-013/NCT01953692 Kuruvilla et al. (2023)	single-arm	Ib	17.9 m (range: 0.8–46.8)	pembrolizumab	r/r DLBCL (ASCT ineligible)	42	n/a	n/a
2022	Carla Casulo	FUSION NHL-001/NCT02733042 Casulo et al. (2023)	single-arm	I/II	23.3 m	durvalumab	r/r DLBCL	10	62	60
2019	Alex F. Herrera	NCT02401048 Herrera et al. (2020)	single-arm	Ib/II	17.5 m (range: 0.2–23.6)	durvalumab+ ibrutinib	r/r DLBCL	34	67	61.8
2019	Anas Younes	LYM1002/NCT02329847 Younes et al. (2019); Younes et al. (2017)	single-arm	I/IIa	18.4 m (IQR: 14.8–19.4)	nivolumab+ ibrutinib	r/r DLBCL	45	64	64
2019	Constantine S. Tam	NCT02795182 Tam et al. (2019)	single-arm	Ib	3 m (range: 0.1–28.3)	tislelizumab+ zanubrutinib	r/r DLBCL	27	65	n/a
2019	Thomas E. Witzig	KEYNOTE-145/NCT02362035 Witzig et al. (2019)	single-arm	I/II	n/a	pembrolizumab+ acalabrutinib	r/r DLBCL	61	67	n/a
2022	L. Zuo	L. Zuo, 2022 Zuo et al. (2022)	single-arm	II	11.1 m	tislelizumab+ zanubrutinib	refractory DLBCL	10	60.5	70
2023	Y. Qin	NCT04425824 Qin et al. (2023)	single-arm	II	19.2 m (95% CI [15.4–25.7])	toripalimab/ nivolumab/ pembrolizumab/ sintilimab+rituximab	r/r DLBCL	26	n/a	n/a
2022	M. Lia Palomba	NCT02220842 Palomba et al. (2022b)	single-arm	Ib	35.9 m (95% CI [35.9–41.2])	atezolizumab+ obinutuzumab	r/r DLBCL	23	69	60.9
2022	Carla Casulo	FUSION NHL-001/NCT02733042 Casulo et al. (2023)	single-arm	I/II	14.8 m	durvalumab+ rituximab+ bendamustine	r/r DLBCL	10	60	60
2021	Eliza A. Hawkes	JAVELIN/NCT02951156 Hawkes et al. (2021)	single-arm	Ib	n/a	avelumab+ rituximab+ bendamustine	r/r DLBCL	11	70	81.8
2022	Max S. Topp	NCT02729896 Topp et al. (2023)	single-arm	Ib	5.7 m (range: 0.9–15.4)	atezolizumab+ obinutuzumab+ polatuzumab vedotin	r/r DLBCL	21	68	62
2021	A. Davies	ARGO/NCT03422523 Davies et al. (2021)	RCT	II	n/a	atezolizumab+ R-GEMOX	r/r DLBCL	41	n/a	n/a
2022	J. Mu	J. Mu, 2022 Mu et al. (2023)	non-randomized controlled	n/a	n/a	sintilimab+ CD19 CAR-T	r/r DLBCL	26	52	69.2
2020	Caron A. Jacobson	ZUMA-6/NCT02926833 Jacobson et al. (2020)	single-arm	I/II	10.2 m	atezolizumab+ axicabtagene ciloleucel	r/r DLBCL	28	58	57.1
2023	Ulrich Jaeger	PORTIA/NCT03630159 Jaeger et al. (2023)	single-arm	Ib	n/a	pembrolizumab+ tisagenlecleucel	r/r DLBCL	12	62	66.7
2020	E. Tholouli	ALEXANDER/ NCT03287817 Tholouli et al. (2020)	single-arm	I	n/a	pembrolizumab+ AUTO3	r/r DLBCL	23	57	n/a
2023	Y. Zhang	NCT 04539444 Zhang et al. (2024)	single-arm	II	n/a	tislelizumab+ CD19/22 dual-targeted CAR-T	r/r DLBCL	13	52	69.2
2020	Charles Herbaux	NCT03276468 Herbaux et al. (2020)	single-arm	II	9 m	atezolizumab+ obinutuzumab+ venetoclax	r/r DLBCL	58	70	53.4
2021	Philippe Armand	CHECKMATE-039/NCT01592370 Armand et al. (2021)	single-arm	Ib	n/a	nivolumab+ ipilimumab	r/r DLBCL	11	n/a	n/a
N/a	ClinicalTrials.gov	NCT02253992 Bristol-Myers Squibb (2020)	single-arm	I/II	n/a	nivolumab+ urelumab	r/r DLBCL	22	n/a	n/a
2022	M. Lia Palomba	NCT02220842 Palomba et al. (2022a)	single-arm	Ib	23.7 m (95% CI [19.8–25.9])	atezolizumab+ tazemetostat	r/r DLBCL	43	65	74.4
N/a	ClinicalTrials.gov	KEYNOTE-037/NCT02178722 Incyte Corporation (2022)	single-arm	I/II	n/a	pembrolizumab+ epacadostat	r/r DLBCL	26	n/a	n/a
2023	Carmelo CarloStella	NCT03769181 Carlo-Stella et al. (2023)	single-arm	I/II	n/a	cemiplimab+ isatuximab	r/r DLBCL	17	64	70.6
2021	Philippe Armand	CHECKMATE-039/NCT01592370 Armand et al. (2021)	single-arm	Ib	n/a	nivolumab + lirilumab	r/r DLBCL	26	n/a	n/a
2022	John Kuruvilla	KEYNOTE-013/NCT01953692 Kuruvilla et al. (2023)	single-arm	Ib	n/a	pembrolizumab+ lenalidomide	r/r DLBCL	19	63.0	57.9
2022	Gareth P. Gregory	KEYNOTE-155/NCT02684617 Gregory et al. (2022)	single-arm	Ib	n/a	pembrolizumab+ dinaciclib	r/r DLBCL	38	64.5	60.5
2021	Vincent Ribrag	NCT02549651 Ribrag et al. (2021)	single-arm	Ib	n/a	durvalumab+ danvatirsen	r/r DLBCL	29	n/a	n/a
2022	Pratyush Giri	NCT03340766 Giri et al. (2022)	single-arm	Ib	n/a	pembrolizumab+ blinatumomab	r/r DLBCL	31	n/a	n/a
2023	Neil L Berinstein	SPIREL/NCT03349450 Amitai et al. (2023)	single-arm	2	10	pembrolizumab+ DPX-Survivac	r/r DLBCL	25	74	44
2023	Armando Santoro	NCT03598608 Santoro et al. (2023)	single-arm	I/II	NA	pembrolizumab+ favezelimab	r/r DLBCL	25	73	n/a

In the only RCT (ARGO/NCT03422523), the 12-month PFS rate of atezolizumab plus R-GEMOX group was 15.2% (95% CI [5.8–28.8]), compared with 8.3% (95% CI [0.51–31.1]) of control R-GEMOX group, and estimated HR was 0.88 (95% CI [0.45–1.73]; *p* = 0.718) (Davies et al., 2021).

#### OS

**Figure 4:**
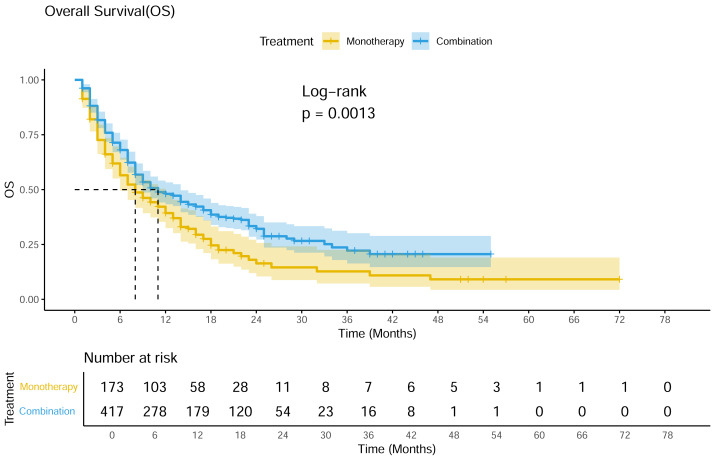
Pooled OS curves of DLBCL patients treated with PD-1/PD-L1 mAb monotherapy and combination therapy.

**Figure 5:**
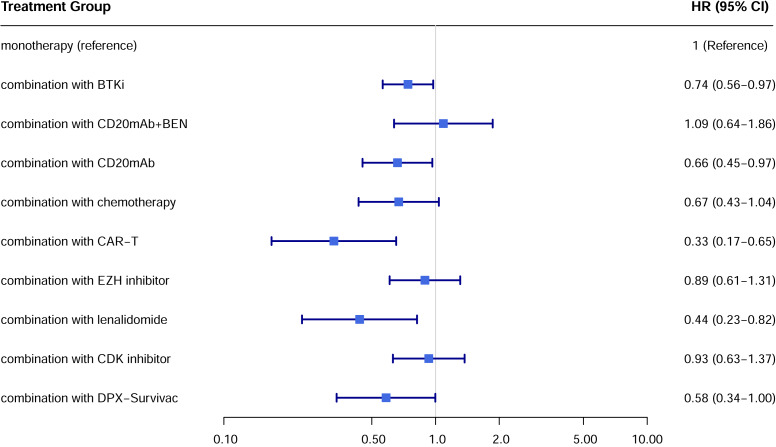
Hazard ratio for OS of each treatment subgroup.

A total of 19 studies showed available OS curves. The pooled curve indicated a better OS of combination therapy compared with monotherapy group (Fig. 4), with a statistical difference (*p* = 0.013) and a HR of 0.71 (95% CI [0.57–0.88]). The 12-month OS rates were 48.0% and 39.3%, and the 24-month OS rates were 32.1% and 16.4%, respectively. Compared with monotherapy, the combination with CAR-T subgroup demonstrated superior survival benefits across all subgroups (HR 0.33; 95% CI [0.17–0.65]) (Fig. 5). In the only RCT (ARGO/NCT03422523), the 12-month OS rate of atezolizumab plus R-GEMOX group was 53.9% (95% CI [37.0–68.1]), *versus* 58.3% (95% CI [27.0–80.1]) for R-GEMOX control group, the estimated HR was 1.26 (95% CI [0.48–3.32]; *p* = 0.642) (Davies et al., 2021).

#### Response rate

**Figure 6:**
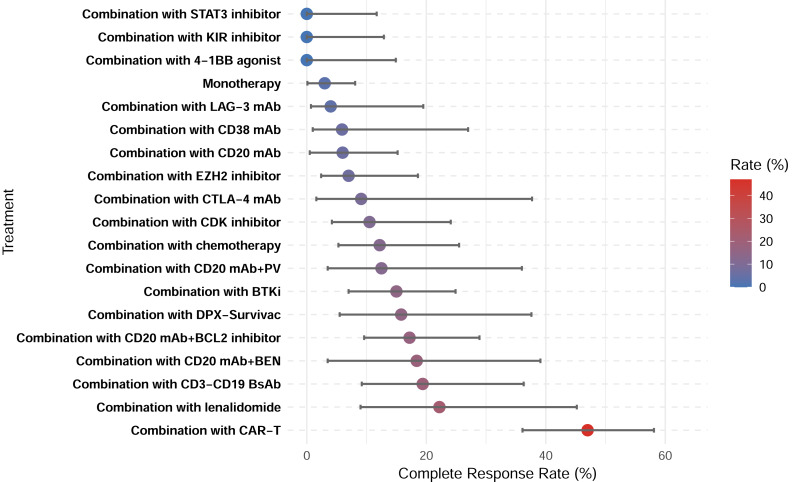
Complete response rates of treatment subgroups.

**Figure 7:**
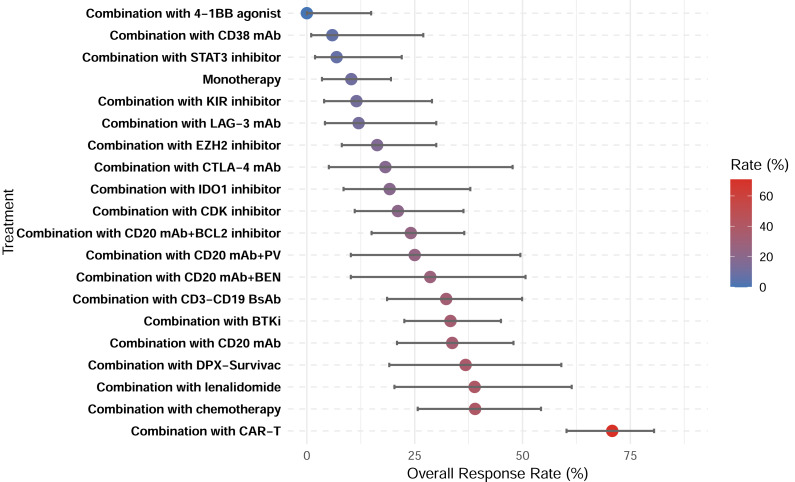
Overall response rates of treatment subgroups.

A total of 28 studies showed CRR, with a pooled CRR of 12.4% (Fig. S2). Subgroup analysis demonstrated significantly higher CRR for combination therapy compared with monotherapy (14.6% *vs.* 3.0%; *p* < 0.001; Figs. S3–S4). Among the 29 studies reporting ORR, the pooled analysis demonstrated an ORR of 27.0% (Fig. S5). Similarly, a higher ORR for combination therapy was demonstrated (30.5% *vs.* 10.3%; *p* < 0.001; Figs. S6–S7). Among all therapeutic subgroups, the CAR-T combination group demonstrated the highest response rate, achieving superior outcomes in both CRR and ORR (Figs. 6–7).

In the only RCT (ARGO/NCT03422523), the ORR of atezolizumab plus R-GEMOX group was 38% (CR 13%), while that of R-GEMOX control group was 27% (CR 9%). No statistical difference in response was observed between the two groups (Davies et al., 2021).

#### Subgroup analysis

##### PD-L1 positive subgroup.

Given the heterogeneity in criteria of PD-L1 positivity across studies, we performed a qualitative synthesis to evaluate the association between PD-L1 expression and treatment outcomes (Table 2).

**Table 2:** Impact of PD-L1 positivity in different studies.

Study	PD-L1 positivity criterion	Relationship between positivity and efficacy
NCT04425824 Qin et al. (2023)	PD-L1 combined positive score ≥ 1%	No significant correlation.
ARGO/NCT03422523 Davies et al. (2021)	Gain of PD-L1 locus at 9p by fluorescence in situ hybridization (FISH)	ORR, PFS (median: 7.2 m *vs.* 3.2 m; *p* = 0.037) and OS (median: not reached *vs.* 5 m; *p* = 0.025) were higher in tumors with gain of PD-L1 locus at 9 p. This was not reflected in immunohistochemistry (IHC).
NCT02650999 Chong et al. (2022)	Total PD-L1 expression through IHC	All responding patients had biopsies between 10–50% total PD-L1 expression.
KEYNOTE-013 Kuruvilla et al. (2023)	Absent (H-score = 0), low(1–99), and high expression (≥100)	ORR of PD-L1 positive group and negative group were 75% (3/4) and 25% (2/8), respectively.
LYM1002 Younes et al. (2019) and Younes et al. (2017)	≥5% positive tumor cells *via* IHC	63% (5/8) of patients with elevated PD-L1 expression achieved response compared with 19% (3/16) for patients without elevated expression (*p* = 0.065). Reportable PD-L1 values were seen in 3/5 CR patients, and all 3 ones had elevated expression of PD-L1.
Mu et al. (2023)	Expression of PD-1 in CD3^+^T-cells using flow cytometry	PD-1 expression was 39.55 ± 11.57% in the CR group, 43.52 ± 12.96% in the partial response (PR) group, and 46.06 ± 6.53% in the non-response group. There was no significant difference in PD-1 expression among the three groups.
NCT02549651 Ribrag et al. (2021)	IHC staining of tumor cells and immune cells. Positivity cut-offs were 1% and 25%.	Of the 2 patients with PR, 1 patient had no PD-L1 expression on tumor cells and 10% expression on immune cells, and 1 patient had no available tumor tissue for analysis.

##### Non-GCB subgroup.

Two studies (NCT02401048: durvalumab plus ibrutinib (Herrera et al., 2020); NCT02795182: tislelizumab plus zanubrutinib (Tam et al., 2019)) reported response rates of the non-germinal center B-cell-like (non-GCB) subgroup, with a pooled CRR of 25.6% and an ORR of 38.7%. A marginally statistical difference was observed in CRR (*p* = 0.056), while no difference was found in ORR when compared with the germinal center B-cell-like (GCB) subgroup. NCT02401048 trial showed available survival curves of GCB and non-GCB subtypes, with an estimated PFS HR of 0.66 (95% CI [0.30–1.44]; *p* = 0.294) of non-GCB compared with GCB subtype (Herrera et al., 2020).

### Safety

**Figure 8:**
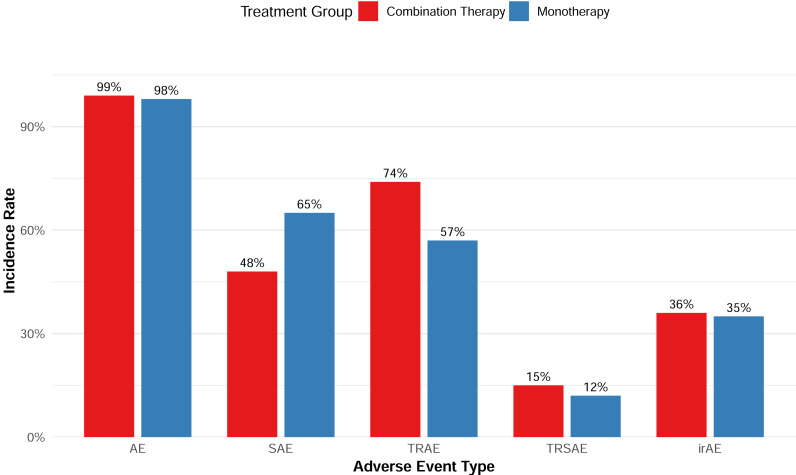
Adverse event rates of combination and monotherapy group.

Overall, the combination therapy demonstrated a manageable safety profile without significantly increasing the incidence of serious adverse events (SAEs) (Fig. 8). In terms of AEs of special interest, six studies reported the incidence of immune-related AEs (irAEs), with a pooled incidence of 35.5%; the cytokine release syndrome (CRS) incidence data reported by seven studies showed a borderline difference between the monotherapy and the combination with CAR-T therapy (*p* = 0.082).

### Publication bias and sensitivity analysis

The monotherapy group exhibited mild publication bias in CRR (Egger’s test *p* = 0.6013; one added study through Trim-and-Fill method) and ORR (Egger’s test *p* = 0.5595; one added study through Trim-and-Fill method) (Fig. S8), with no publication bias in the incidences of AEs (Egger’s test *p* = 0.8843) and SAEs (Egger’s test *p* = 0.5712) (Figs. S10–S12). However, evidence of significant publication bias could be found in the combination treatment group. Trim-and-Fill adjusted CRR (Egger’s test *p* = 0.0008; Begg’s test *p* = 0.02; seven added study through Trim-and-Fill method) and ORR (Egger’s test *p* = 0.2043; Begg’s test *p* = 0.2763; three added study through Trim-and-Fill method) were 21.2% (95% CI [15.3–28.6]) and 34.4% (95% CI [26.5–43.2]) (Fig. S9), respectively. There was also publication bias in the incidence of AEs (Egger’s test *p* = 0.0156; Begg’s test *p* = 0.2165), the Trim-and-Fill adjusted incidence was 91.0% (95% CI [87.6–93.5]) (Fig. S11). Sensitivity analysis confirmed the robustness of the findings.

## Discussion

Based on our meta-analysis, the inferior response rate and survival of PD-1/PD-L1 mAb monotherapy in DLBCL could not support its application in unselected population. Although DLBCL exhibits relatively high PD-L1 expression compared with other NHL subtypes, the expressions of PD-1 and PD-L1 are usually heterogeneous (PD-1: 39.5%-68.6%; PD-L1: 25–70% (Xu-Monette, Zhou & Young, 2018)), and PD-L1 structural variations (SVs) can be identified through FISH in only 20–25% of DLBCL patients (more common in non-GCB subtype) (Godfrey et al., 2019). In addition, a majority of DLBCL cases can be classified as “non-inflammatory lymphoma”, characterized by the absence of immune-related gene expression and the presence of immune escape mechanisms, especially in the GCB subtype (Kline, Godfrey & Ansell, 2020). The aforementioned mechanisms may account for the limited efficacy of PD-1/PD-L1 mAb monotherapy in DLBCL, yet there is still no reliable predictive markers. Although patients of non-GCB subtype may benefit more from PD-1/PD-L1 mAb, unfortunately, a small number of studies on the non-GCB subtype only showed modest efficacy (ORR 37.5%–40%), the potential therapeutic benefits for the non-GCB subtype still require more verification. In a retrospective analysis of KEYNOTE-013 study, a significant correlation was reported between PD-L1 SVs and the ORR of pembrolizumab (Godfrey et al., 2019). In order to validate the predictive significance of PD-L1 SVs, a phase II trial (NCT03990961) of r/r DLBCL patients with PD-L1 gene change is undergoing. Therefore, the next direction of PD-1/PD-L1 mAb in DLBCL should be identifying reliable predictive markers and exploring combination strategies. While our analysis focused exclusively on DLBCL, it is worth noting that PD-1/PD-L1–based therapies have demonstrated distinct efficacy profiles in other malignancies, particularly solid tumors (Ferris et al., 2016; Chen et al., 2021; Xie et al., 2025). However, due to the substantial biological heterogeneity between DLBCL and other cancers (Chen et al., 2025), direct extrapolation of our findings is not appropriate. Broader cross-cancer analyses would be valuable to further understand the mechanistic basis of differential responses (Lee & Ruppin, 2019; Kline, Godfrey & Ansell, 2020).

Chemotherapy can trigger immunogenic death of tumor cells, and released immunogenic signals can trigger immune response, thereby eliminating or controlling residual tumor cells. However, the efficacy of atezolizumab plus R-GEMOX was not satisfactory (CR 13%, ORR 38%) (Davies et al., 2021). Ibrutinib can inhibit regulatory T-cells (Tregs), promote the activation of cytotoxic T-cells (CTLs) and enhance the secretion of inflammatory cytokines (Gopal et al., 2018), expected to provide a more favorable tumor microenvironment (TME) for PD-1/PD-L1 mAb. However, the ORRs (23.5–36%) of durvalumab plus ibrutinib (NCT02401048) (Herrera et al., 2020) and nivolumab plus ibrutinib (LYM1002/NCT02329847) (Younes et al., 2019; Younes et al., 2017) regimens were similar to that of ibrutinib monotherapy, even with additional toxicities (Winter et al., 2017). Nuclear factor kB (NF-kB) activation is often enhanced in the inflammatory DLBCL subgroup with PD-L1 SVs (Godfrey et al., 2019), however, the combination of pembrolizumab plus lenalidomide did not exhibit an obvious synergistic effect, the ORR in KEYNOTE-013 study was similar to that of lenalidomide monotherapy in ReMIND study (39% *vs.* 34%) (Zinzani et al., 2021).

Effects of CD20 mAb include complement-dependent cytotoxicity (CDC), antibody-dependent cellular cytotoxicity (ADCC), antibody-dependent cellular phagocytosis (ADCP), and direct apoptosis induction (Pavlasova & Mraz, 2020). Regrettably, atezolizumab plus obinutuzumab (NCT02220842) (Palomba et al., 2022b) and the triple combination of atezolizumab, obinutuzumab, and venetoclax (NCT03276468) (Herbaux et al., 2020) regimens only showed limited activity in r/r DLBCL patients (ORR 17.4%–23.6%). The safety of other novel agents targeting tumor-antigen, including ADC and BsAb, remain a concern when combined with PD-1/PD-L1 mAb. Combination of atezolizumab, obinutuzumab, and polatuzumab vedotin (an ADC targetting CD79b) induced severe AEs but limited activity, leading to an early termination of the study (Topp et al., 2023). NCT03340766 study exploring blinatumomab (a CD3-CD19 dual antibody) combined with pembrolizumab was also prematurely terminated due to little therapeutic benefit (Giri et al., 2022).

Accumulation of epigenetic modifications during tumor development promotes immune escape, while epigenetic regulatory factors can influence the TME of DLBCL (Banik, Moufarrij & Villagra, 2019). Although the safety of atezolizumab plus tazemetostat (an inhibitor of enhancer of zeste homolog 2 [EZH]) was tolerable, the response rate and long-term survival were not satisfactory (Palomba et al., 2022a). Additionally, in a case series of seven DLBCL patients who progressed after CAR-T and subsequently received chidamide (a histone deacetylase inhibitor [HDACi]) plus sintilimab, two patients achieved CR, and CR patients were not PD-L1 over-expressed (Hao et al., 2022). However, there is still a lack of large size study on the combination of HDACi.

CAR-T treatment achieved a remarkable response rate in post-second-line treatment of r/r aggressive B-cell lymphomas (Abramson et al., 2020; Schuster et al., 2019), however, disease relapse occurs in a proportion of cases, leading to a poor prognosis (Locke et al., 2019). CAR-T failure may be related to an immunosuppressive TME and CAR-T depletion induced by immune checkpoints (Kasakovski, Xu & Li, 2018). Therefore, the combination of CAR-T with immune checkpoint inhibitors (ICIs) represents a rational approach. Although this regimen demonstrated superior PFS, current clinical data have not shown an extra benefit compared with CAR-T monotherapy. Due to the inadequate follow-up time, no sufficient evidence could prove the long-term prognosis benefits, thus this combination strategy requires further cautious evaluation due to its potential to exacerbate the treatment burden. Therapeutic option is more limited for patients with disease progression post CAR-T therapy, while ICIs may be a viable salvage option. In a small size study, pembrolizumab could amplify and reactivate the depleted CAR-T cells in patients after the treatment failure of CD19 CAR-T, and one more times of CAR-T expansion could lead to a second response (Chong et al., 2022). These findings suggest that ICIs may still provide additional benefits for patients who received CAR-T therapy. Notably, although the combination of PD-1/PD-L1 blockade with CAR-T therapy demonstrated the most favorable survival outcomes in our analysis, its clinical applicability is constrained by cost, manufacturing complexity, and limited availability, particularly in developing regions. This paradox highlights that while PD-1/PD-L1 plus CAR-T represents a highly promising strategy, its broad application remains challenging, and alternative partner agents for PD-1/PD-L1 blockade should continue to be explored in parallel.

Beyond PD-1/PD-L1, immune cells express multiple co-inhibitory/co-stimulatory molecules. Theoretically, PD-1/PD-L1 mAb could be combined with other immune modulators to enhance the anti-tumor immunity by simultaneously blocking different immune checkpoints: (1) Cytotoxic T-lymphocyte associated protein 4 (CTLA-4) is another key co-inhibitory molecule that negatively regulates immunity, and ipilimumab revealed rare but persistent response in DLBCL patients in a phase I study (Ansell et al., 2009), however, the efficacy of nivolumab plus ipilimumab in DLBCL was disappointing (Armand et al., 2021), with no difference in response compared with PD-1 mAb monotherapy, and a significantly increased incidence of irAEs; (2) Agonist of co-stimulatory receptor 4-1BB (CD137) may generate synergistic effects with PD-1/PD-L1 blockade, however, the result of nivolumab plus urelumab (NCT02253992) was disappointing (ORR=0) (Bristol-Myers Squibb, 2020); (3) Indoleamine 2,3-dioxygenase (IDO) can suppress multiple immune cells, but pembrolizumab plus epacadostat only showed modest efficacy (ORR 19.2%) (Incyte Corporation, 2022); (4) Acquired resistance of PD-1/PD-L1 mAb is associated with CD8^+^T-cell inhibition mediated by upregulated CD38, however, no synergistic effect between isatuximab and cemiplimab was observed in DLBCL (Carlo-Stella et al., 2023); (5) The interaction between killer cell immunoglobulin like receptor (KIR) and human leukocyte antigen (HLA) mediates a cytotoxic tolerance to NK cells, while the combination of lirilumab and PD-1 mAb only achieved a ORR of 11.5% (Armand et al., 2021). Despite the mechanistic rationales, the majority of combination therapies involving PD-1/PD-L1 blockade and additional immune modulators have demonstrated limited clinical efficacy.

Additionally, DLBCL is a molecularly heterogeneous disease (Scherer et al., 2016), and molecular subtype provides a rationale for the precise treatment. For instance, the MCD subtype (MYD88 and CD79B mutations) exhibits active immune evasion mechanisms, which makes them compelling candidates for enhanced responses to ICIs (Wright et al., 2020). The genomic characterization on the liquid biopsy combined with radiomics may help to stratify and identify patients who are candidates to combination treatments (Dondolin et al., 2025), such approaches represent a promising avenue for precision medicine in r/r DLBCL.

The limitation of this meta-analysis was the small number of corresponding studies or total sample size of some regimens. Most eligible studies were single-arm trials, so there might be patients heterogeneity. Although some combinations achieved promising results, large-scale controlled studies are still needed to determine the value of PD-1/PD-L1 mAb to their partner therapies.

## Conclusion

PD-1/PD-L1 mAb monotherapy is generally tolerable but with limited activity in r/r DLBCL. Combination therapies demonstrated superior response rate and survival without increased toxicities, although these findings were derived from cross comparisons of single-arm studies. Further research should prioritize the identification of predictive biomarkers and patient subgroups more likely to benefit.

##  Supplemental Information

10.7717/peerj.20314/supp-1 Supplemental Information 1 Supplementary information

10.7717/peerj.20314/supp-2 Supplemental Information 2 Raw Data

10.7717/peerj.20314/supp-3 Supplemental Information 3 Intended Audience

10.7717/peerj.20314/supp-4 Supplemental Information 4 PRISMA checklist

## Abbreviations

PD-1/PD-L1: programmed death 1/programmed death ligand 1
mAb: monoclonal antibody
r/r: relapsed/refractory
DLBCL: diffuse large B-cell lymphoma
PFS: progression-free survival
OS: overall survival
CRR: complete response rate
ORR: overall response rate
AEs: adverse events
NHL: non-Hodgkin lymphoma
CR: complete response
ASCT: autologous stem cell transplantation
CAR-T: chimeric antigen receptor T-cell
ADC: antibody-drug conjugate
BsAb: bispecific antibody
BCL2: B-cell lymphoma 2
BTKi: Bruton’s tyrosine kinase inhibitor
PD-L2: programmed death ligand 2
ITIM: immunoreceptor tyrosine based inhibition motif
ITSM: immunoreceptor tyrosine based switch motif
SRC: SRC proto-oncogene
PI3K: phosphatidylinositol-3-kinase
mAbs: monoclonal antibodies
DLBCL NOS: DLBCL, not otherwise specified
PMBL: primary mediastinal large B-cell lymphoma
RCT: randomized controlled studies
MINORS: methodological index for non-randomized studies
KM: Kaplan–Meier
CI: confidence intervals
HR: hazard ratio
FISH: fluorescence in situ hybridization
IHC: Immunohistochemistry
non-GCB: non-germinal center B-cell- like
GCB: germinal center B-cell- like
SAEs: serious adverse events
irAEs: immune-related AEs
CRS: cytokine release syndrome
SVs: structural variations
HGBL: high-grade B-cell lymphoma
Tregs: regulatory T-cells
CTLs: cytotoxic T-cells
CDC: complement-dependent cytotoxicity
ADCC: antibody-dependent cellular cytotoxicity
ADCP: antibody-dependent cellular phagocytosis
EZH2: enhancer of zeste homolog 2
HLA: human leukocyte antigen
HDACis: histone deacetylase inhibitors
ICIs: immune checkpoint inhibitors
CTLA-4: cytotoxic T-lymphocyte associated protein 4
IDO: indoleamine 2,3-dioxygenase
KIR: killer cell immunoglobulin like receptor
NF-kB: nuclear factor kB
STAT3: signal transducer and activator of transcription 3
TME: tumor microenvironment

## Additional Information and Declarations

### Data Availability

The following information was supplied regarding data availability: This is a systematic review/meta-analysis.
