# Supplementary material for: Comparison of anti-PD-1/PD-L1-based regimens in relapsed/refractory diffuse large B-cell lymphoma: a meta-analysis
Source: PeerJ. 2025 Nov 19;13:e20314. doi: 10.7717/peerj.20314 (PMC12640133; doi:10.7717/peerj.20314)
Supplement: Supplemental Information 1 [file peerj-13-20314-s001.pdf]

Supplementary Information

Supplementary Figure 1: Cochrane risk bias assessment tool for ARGO/NCT03422523

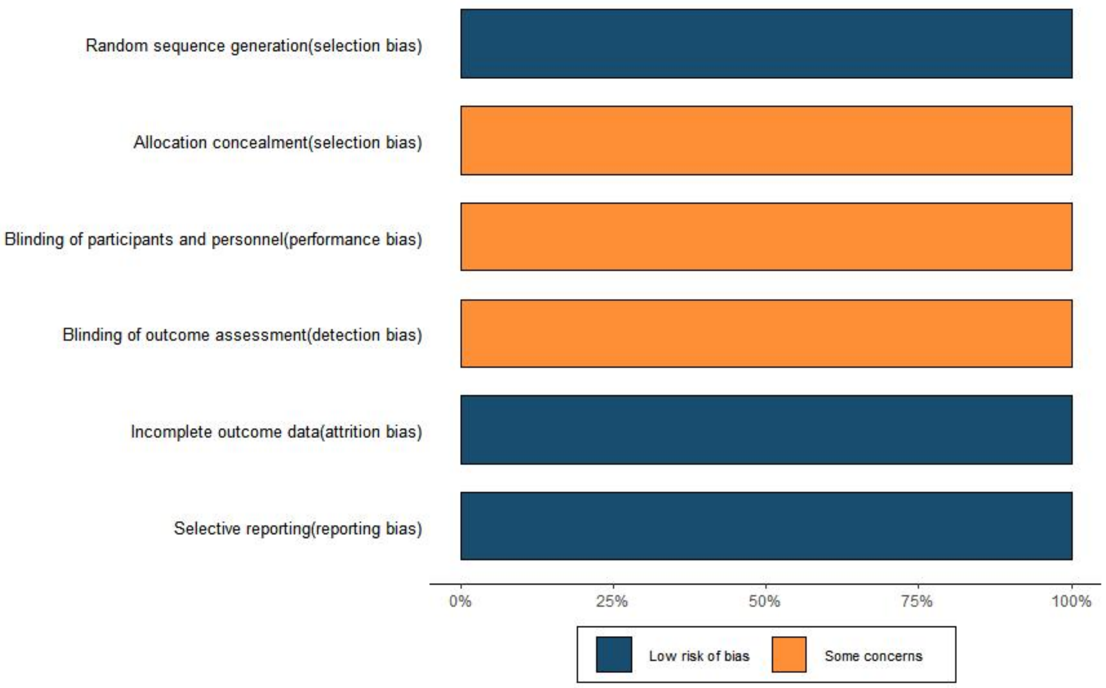

Supplementary Figure 2: pooled complete response rate of PD-1/PD-L1 mAb therapy in DLBCL-NOS

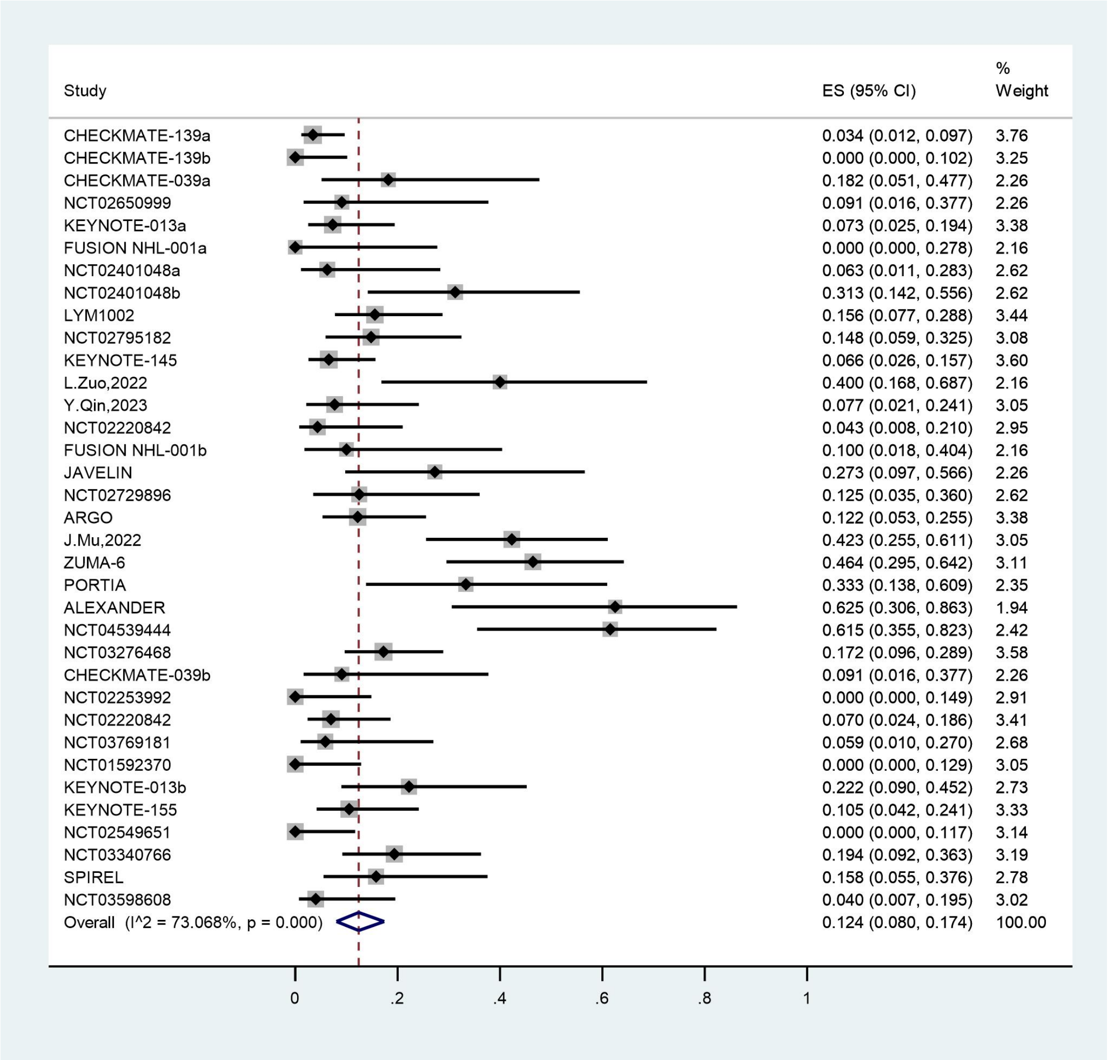

Supplementary Figure 3: pooled complete response rate of PD-1/PD-L1 mAb monotherapy in DLBCL-NOS

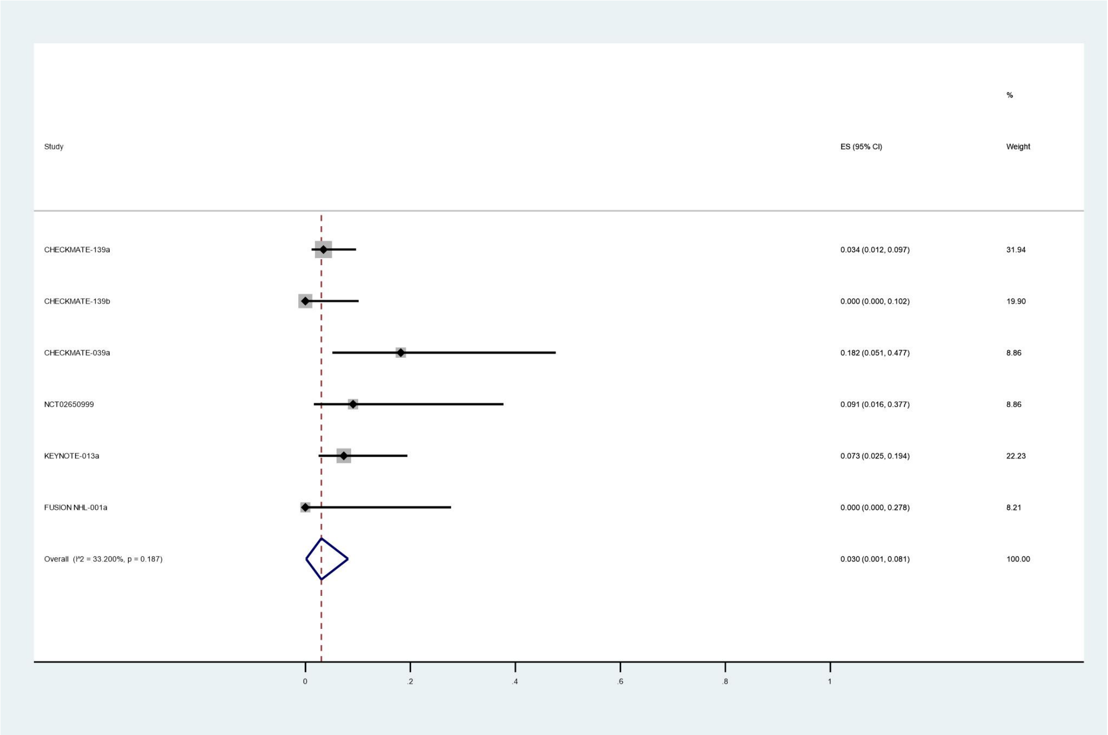

Supplementary Figure 4: pooled complete response rate of PD-1/PD-L1 mAb combination therapy in DLBCL-NOS

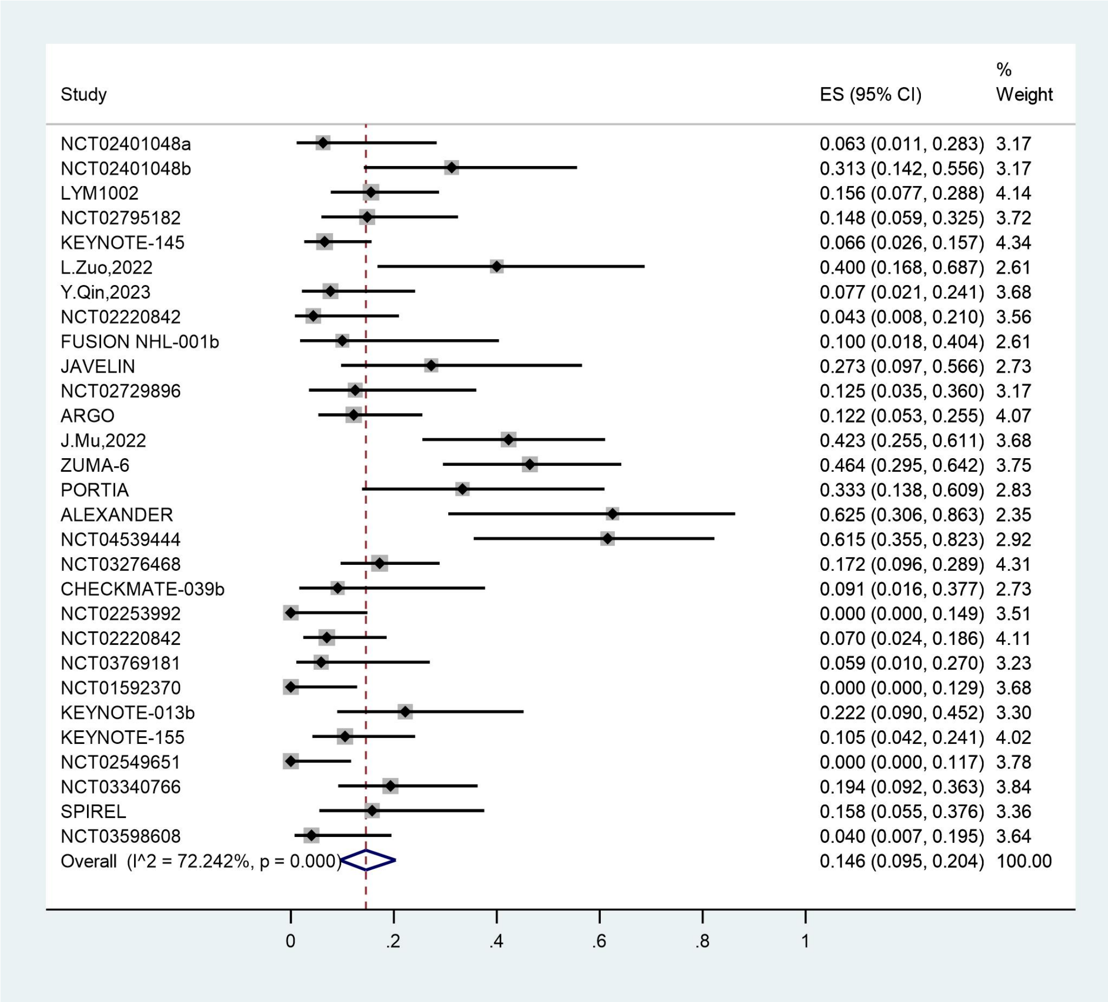

Supplementary Figure 5: pooled overall response rate of PD-1/PD-L1 mAb therapy in DLBCL-NOS

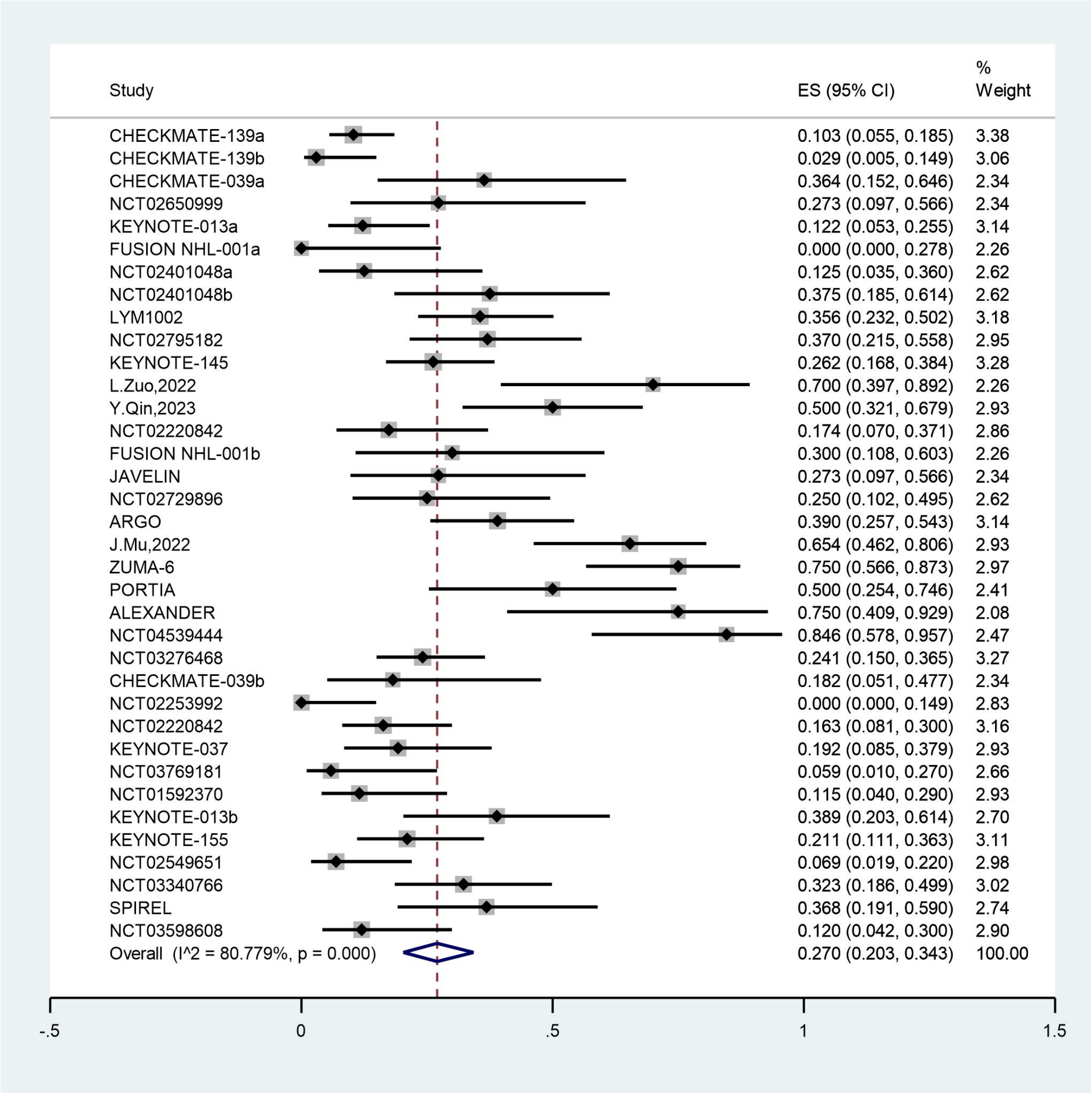

Supplementary Figure 6: pooled overall response rate of PD-1/PD-L1 mAb monotherapy in DLBCL-NOS

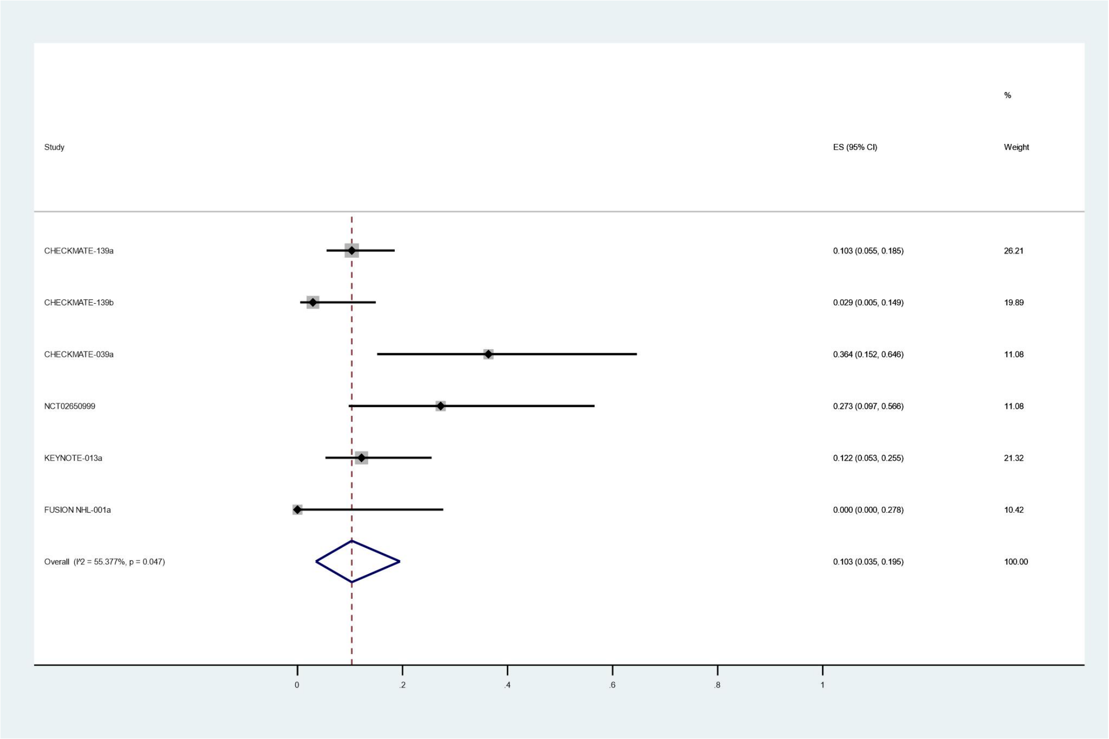

Supplementary Figure 7: pooled overall response rate of PD-1/PD-L1 mAb combination therapy in DLBCL-NOS

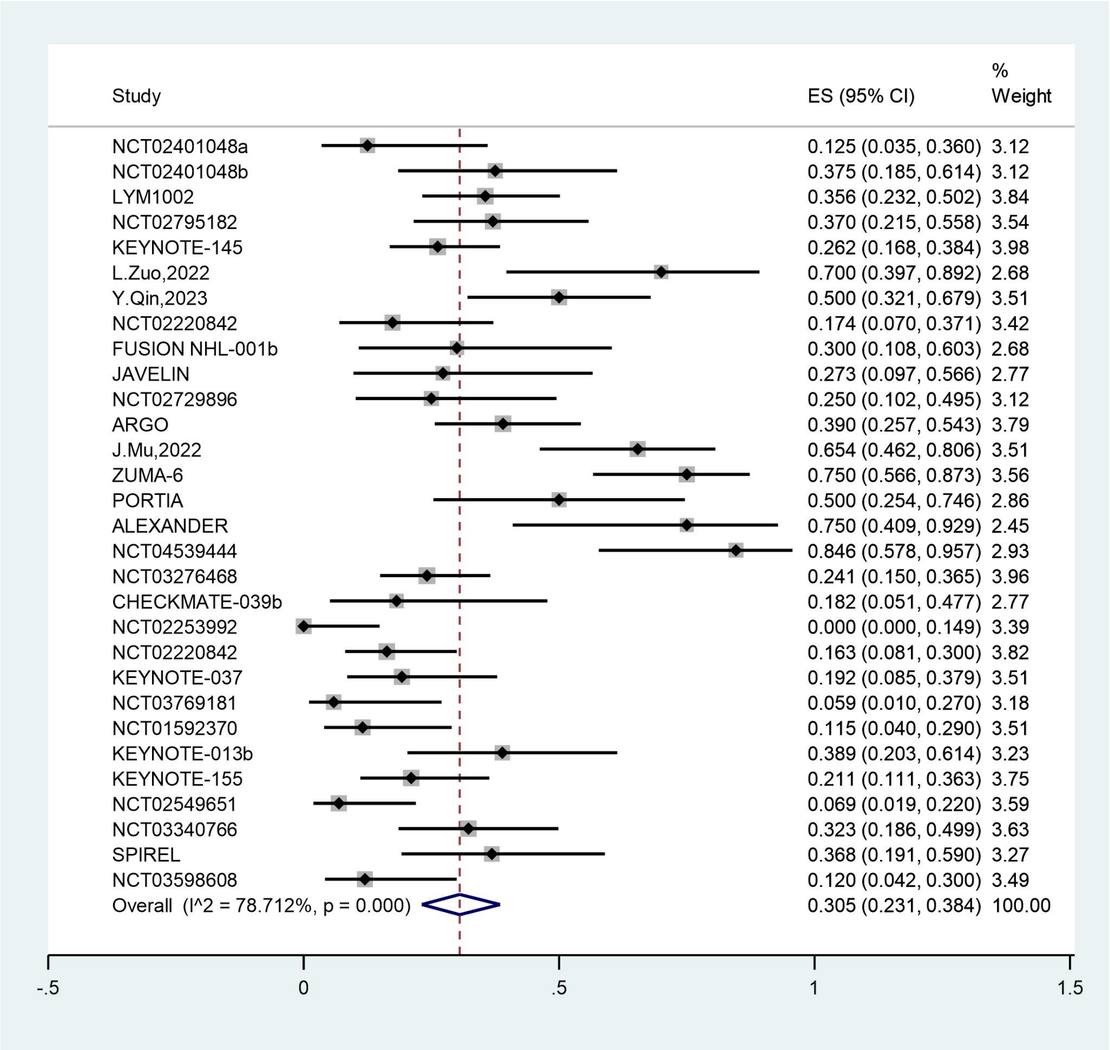

Supplementary Figure 8: Trim-and-Fill adjusted funnel plot for ORR in monotherapy group

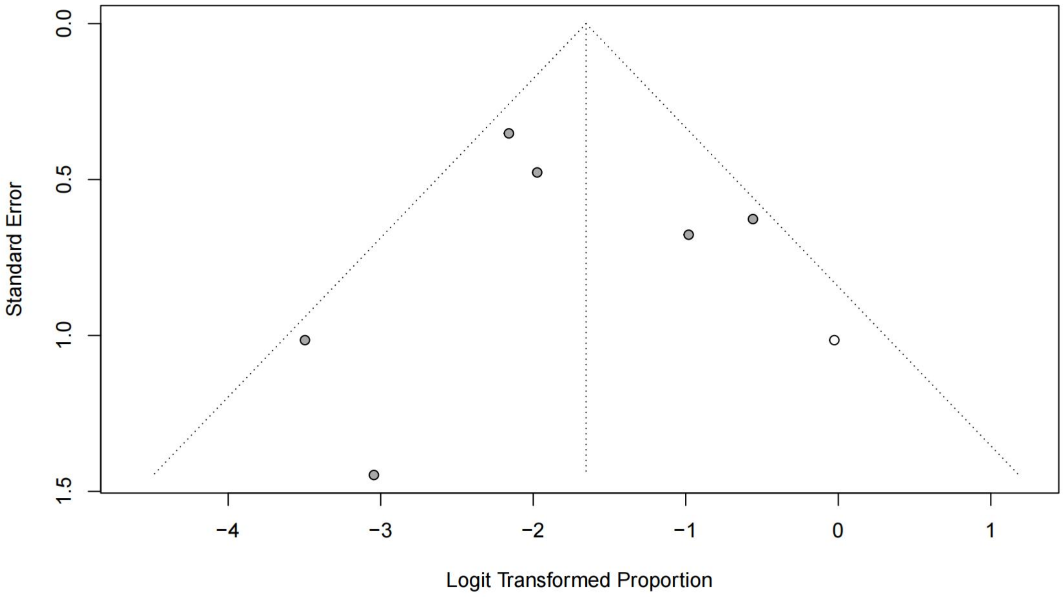

Supplementary Figure 9: Trim-and-Fill adjusted funnel plot for ORR in combination group

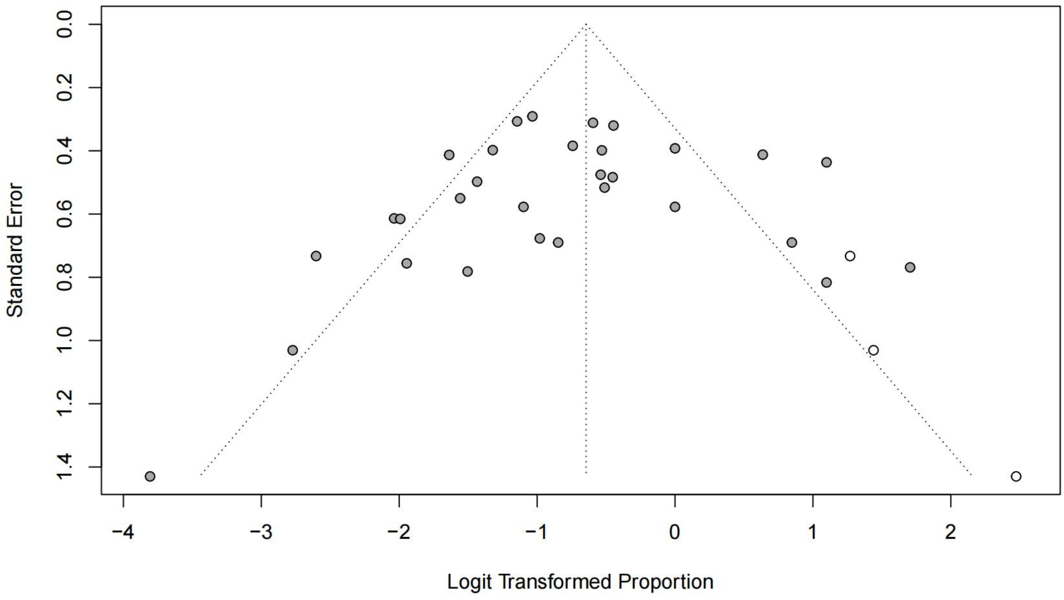

Supplementary Figure 10: Funnel plot for incidence of AEs in monotherapy group

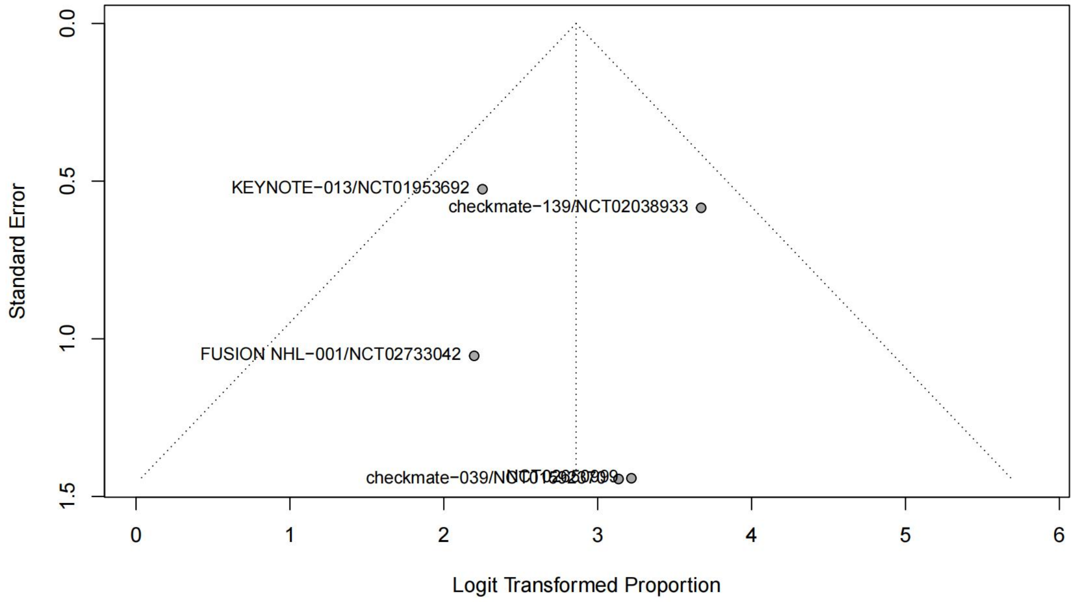

Supplementary Figure 11: Trim-and-Fill adjusted funnel plot for incidence of AEs in combination group

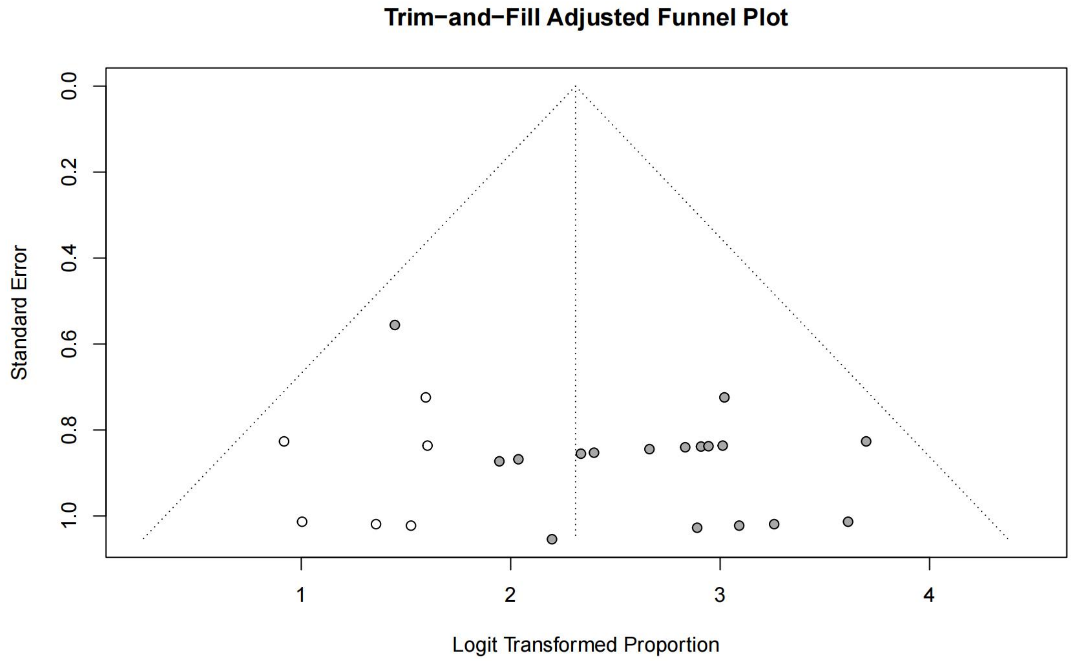

Supplementary Figure 12: Funnel plot for incidence of SAEs in monotherapy group

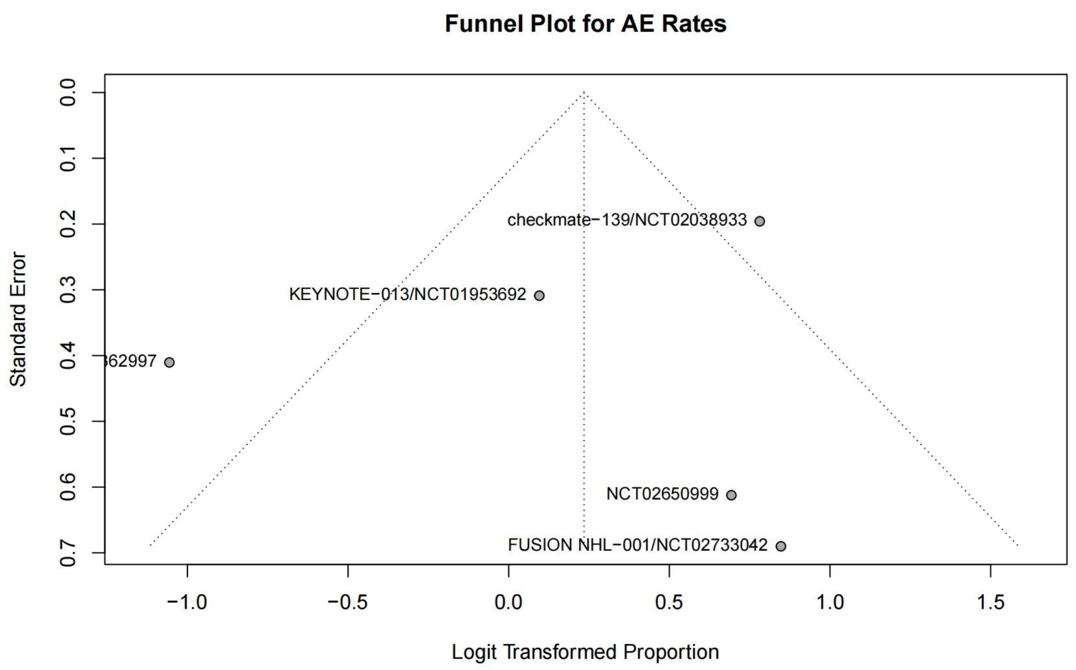

Supplementary Figure 13: Funnel plot for incidence of SAEs in combination group

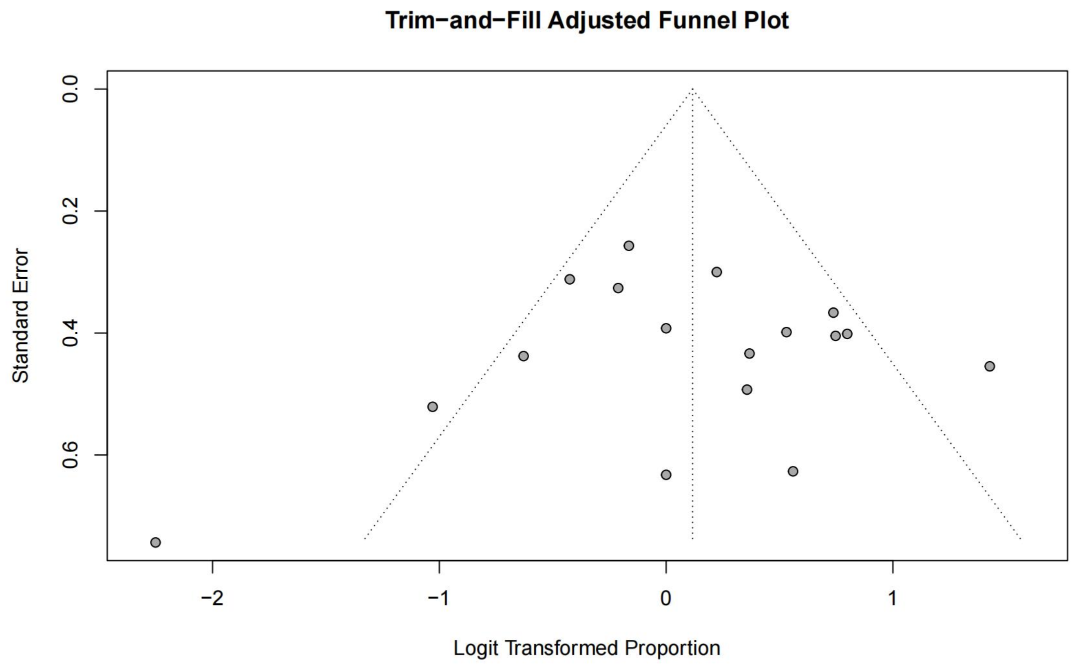

Supplementary Table 1: Methodological index for non-randomized studies (MINORS) for single-arm and non-randomized studies

study	A clearly stated aim	Inclusion of	Prospective collection	Endpoints appropriate	Unbiased assessment	Follow-up period	Loss to follow up less than	Prospective calculation	Total

		consecutive patients	of data	to the aim of the study	of the study endpoint	appropriate to the aim of the study	5%	of the study size	
CHECKMATE-139/NCT02038933	2	2	2	2	2	2	2	2	16
CHECKMATE-039/NCT01592370[a]	2	2	2	2	1	2	2	1	14
NCT02650999	2	2	2	2	1	1	2	1	13
KEYNOTE-013/NCT01953692[a]	2	2	2	2	1	2	2	2	15
FUSION NHL-001/NCT02733042[a]	2	2	2	2	1	2	2	2	15
NCT02401048	2	2	2	2	1	1	2	2	14
LYM1002/NCT02329847	2	2	2	2	1	2	2	2	15
NCT02795182	2	2	2	2	1	2	2	1	14
KEYNOTE-145/NCT02362035	2	2	2	2	1	1	2	1	13
L. Zuo,2022	2	2	2	2	1	2	2	1	14
Y.Qin,2023	2	2	2	2	1	2	2	1	14
NCT02220842[a]	2	2	2	2	1	2	2	1	14
FUSION NHL-001/NCT02733042[b]	2	2	2	2	1	2	2	2	15
JAVELIN/NCT02951156	2	2	2	2	1	1	2	1	13
NCT02729896	2	2	2	2	1	2	2	1	14
J.Mu,2022	2	2	2	2	1	1	2	1	13
ZUMA-6/NCT02926833	2	2	2	2	1	2	1	1	13
PORTIA/NCT03630159	2	2	2	2	1	1	2	1	13
ALEXANDER/NCT03287817	2	2	2	2	1	1	2	1	13
NCT 04539444	2	2	2	2	1	2	2	1	14
NCT03276468	2	2	2	2	1	2	2	1	14
CHECKMATE-039/NCT01592370[b]	2	2	2	2	1	1	2	2	14
NCT02253992	2	2	2	2	1	1	2	2	14
NCT02220842[b]	2	2	2	2	2	2	2	1	15

KEYNOTE-037/ NCT02178722	2	2	2	2	1	1	2	1	13
NCT03769181	2	2	2	2	1	1	2	1	13
CHECKMATE-039/NCT01592370[c]	2	2	2	2	1	1	2	2	14
KEYNOTE-013/ NCT01953692[b]	2	2	2	2	1	1	2	2	14
KEYNOTE-155/ NCT02684617	2	2	2	2	1	1	2	2	14
NCT02549651	2	2	2	2	1	1	2	2	14
NCT03340766	2	2	2	2	1	1	2	1	13
SPIREL/NCT03349450	2	2	2	2	1	2	2	2	15
NCT03598608	2	2	2	2	1	2	2	1	14
