# Supplementary material for: Comparison of anti-PD-1/PD-L1-based regimens in relapsed/refractory diffuse large B-cell lymphoma: a meta-analysis
Source: PeerJ. 2025 Nov 19;13:e20314. doi: 10.7717/peerj.20314 (PMC12640133; doi:10.7717/peerj.20314)
Supplement: Supplemental Information 3 [file peerj-13-20314-s003.docx]

Intended Audience

- ****Hematologists/Oncologists****: Clinicians and researchers specializing in the treatment and management of diffuse large B-cell lymphoma (DLBCL)
- ****Immunotherapy/Immunology Researchers****: Scientists investigating immune checkpoint inhibitors and combination strategies
- ****Evidence-Based Medicine Specialists****: Methodologists and clinicians interested in systematic reviews and meta-analyses of cancer therapeutics
